# Validation of the Nepalese Version of the Quality of Recovery-15 Questionnaire in Patients Undergoing Elective Surgery

**DOI:** 10.31729/jnma.9163

**Published:** 2025-07-31

**Authors:** Chitra Thapa, Sabin Gauchan, Sulav Acharya, Mohan Raj Sharma

**Affiliations:** 1 Department of Anesthesiology, Nepal Medical College and Teaching Hospital, Jorpati, Kathmandu, Nepal

**Keywords:** *elective surgery*, *nepalese*, *quality of recovery-15*, *validation*

## Abstract

**Introduction:**

The 15-item Quality of Recovery scale is a widely used tool for assessing postoperative recovery. It has been translated and validated in various languages and cultural settings. However, a validated Nepalese version is currently unavailable.

**Methods:**

A cross-sectional study was conducted among 216 patients undergoing elective surgery under general anesthesia, following ethical approval (Ref. No. 21-081/082). The 15 items of the Quality of Recovery scale were translated into Nepalese and modified as needed, resulting in the final version: Quality of Recovery scale-N. Patients were interviewed preoperatively (the day before surgery) and on the first postoperative day using the QoR-N. Reliability, validity, responsiveness, and feasibility of the QoR-N were then evaluated.

**Results:**

The Quality of Recovery scale-N showed acceptable reliability, with a Cronbach’s alpha of 0.890, mean inter-item correlation of 0.413, split-half reliability of 0.94, and standard error of measurement of 2.79. Responsiveness was supported by a Cohen’s effect size of 1.4 and standardized response mean of 1.39. QoR-N scores did not correlate with age or surgery duration, and showed no significant difference between ASA physical status I and II, or across minor, intermediate, and major surgeries. However, scores were significantly higher in males than females, and in patients with good overall recovery (per visual analogue scale) compared to those with poor recovery. The recruitment rate was 100%, and completion rate was 94.33%.

**Conclusions:**

The Quality of Recovery scale-N demonstrated acceptable validity, reliability, responsiveness, and clinical feasibility, and is suitable for use in Nepalese patients undergoing elective surgery.

## INTRODUCTION

The process of postoperative recovery includes the patient’s physical, psychological, emotional, and functional well-being.^1^ Patient-reported outcome measures (PROMs) are particularly useful in surgical and anesthetic research, where interventions often aim to improve the patient’s overall postoperative experience.^2^ Among various PROMs developed, the Quality of Recovery-15 (QoR-15) has gained widespread acceptance due to its concise structure, ease of use, and strong psychometric performance.^3-7^

Despite the increasing volume of surgical procedures being performed in our country, there remains a significant gap in the use of standardized PROMs to assess postoperative recovery. Tools like the QoR-15 have not yet been translated, culturally adapted, or validated for use in Nepali-speaking populations.

This study was conducted to translate the Quality of Recovery-15 questionnaire into Nepali language and validate it so that clinicians and researchers can assess recovery quality more accurately and contribute to improved patient care, surgical outcome monitoring, and evidence-based practice.

## METHODS

A cross-sectional observational study was conducted among patients undergoing elective surgery under general anesthesia at Nepal Medical College and Teaching Hospital from 1 September 2024 to 31 March 2025, after receiving ethical approval from the Institutional Review Committee (Reference number: 21-081/082). All patients aged 18 years or above, with American Society of Anesthesiologists (ASA) Physical Status Grade I, II, or III, who could understand and speak Nepali and provided informed consent, were included. Patients with cognitive impairment, those undergoing ambulatory surgery, or those requiring planned or unplanned postoperative transfer to the intensive care unit were excluded.

Permission for translation and validation of the QoR-15 scale was obtained from the original author, Paul Myles, via email. The questionnaire was first translated into Nepali by two native speakers and then back-translated into English by two different bilingual individuals. A panel of four senior anesthesiologists reviewed the translated version. Following minor modifications, a pilot version of the Nepali QoR-15 (QoR-N) was developed and tested on 25 patients undergoing elective inpatient surgeries under general anesthesia, approximately 24 hours postoperatively.

Based on feedback from the pilot testing, items not relevant to the Nepali context were removed, necessary items were added, and the final Nepali version of the Quality of Recovery questionnaire (QoR-N) was established.

The sample size was calculated using the standard “rule of thumb” method, requiring 15 respondents per item.^7^ For 12 items, a minimum of 180 participants was required. To account for a potential 20% dropout rate (due to withdrawal of consent or ICU transfer), the final sample size was set at 216. Convenience sampling was done.

During preanesthetic assessment, eligible patients were counseled about the study. If willing to participate, an informed written consent was obtained from all the participants. Patient particulars such as age, gender, ASA physical status grade, diagnosis, type of surgical procedure to be performed was noted. First QOR-N questionnaire was filled a day before surgery (preoperative QoR-N). The questionnaire had 12 items. An 11-point numerical rating scale was constructed (for positive items i.e. item 1 to item 7, 0= “none of the time” to 10= “all the time”; the scoring was reversed for negative items ie item 8 to item 12, the 0= “all the time” to 10= “none of the time” The minimum possible score of QoR-N was 0 and maximum possible score was 120. Depending upon the patient’s choice, the questionnaire was either read out to them by resident performing pre-anesthetic assessment or they themselves read and filled the form. Patients were followed up after approximately 24 hours in the respective surgical ward. The duration of surgery, as well as any intraoperative and postoperative complications that occurred within the first 24 hours, were noted from the anesthesia and surgical records. The second QoR-N questionnaire (postoperative QoR-N) was repeated. All postoperative interviews were performed by the primary investigator. The questions were read to the patient, and their response was recorded. Additionally, patients were asked to score their overall recovery in a 100 mm visual analogue scale (VAS), marked from 0 ‘poor recovery’ to 100 ‘excellent recovery’.

Data were analyzed in IBM SPSS Statistics for Windows, version 21 (IBM Corp., Armonk, N.Y., USA). The extent of surgery was classified as minor, intermediate, or major depending on the type of surgical procedure and the expected surgical stress response.^8^ The type of surgery was classified according to the surgical subspecialty. Patients were categorized into “good recovery” and “poor recovery” based on VAS score for overall recovery, with VAS ≥70mm categorized in “good recovery” and patients with VAS <70mm categorized in “poor recovery”.

For missing responses, a single missing item was imputed with the worst possible score (0); if two or more items were missing, the QoR-N score was considered invalid.

Clinical feasibility was assessed by evaluating recruitment and completion rates, and by identifying floor and ceiling effects (defined as >15% of participants scoring the lowest or highest possible score).^9^ Normal distribution was assessed with the Shapiro-Wilk test. Changes in the preoperative and postoperative QoR-N were compared by the paired t-test.

Reliability was assessed using internal consistency (Cronbach’s alpha), with a value of ≥0.70 considered acceptable.^10^ The mean inter-item correlation was calculated to assess item homogeneity, with an acceptable range of 0.15 to 0.50.^11^ Split-half reliability was evaluated using the Spearman-Brown coefficient, with values ≥0.70 indicating acceptable reliability.^10^ The Standard Error of Measurement (SEM) was calculated using the formula: SEM SD × square root of 1-α. ^12^

Construct Validity was assessed by evaluating the association between the postoperative QoR-N total score and a global VAS score for overall recovery. A moderate to strong positive correlation was considered evidence of good construct validity.

Criterion validity was examined by assessing the relationships between the postoperative QoR-N score and relevant clinical and demographic variables, including age, gender, ASA-PS of patients, duration of surgery, extent of surgery. Association was tested using Spearman’s rank correlation coefficient for nominal and ordinal variables, and Mann-Whitney U test or Kruskal-Wallis test for group comparisons, as appropriate. A p value of less than 0.05 was considered significant. The correlation of postoperative QoR-N scores with items of physical and mental well being was also analyzed to support the criterion validity of questionnaire.

Structural validity was assessed using exploratory factor analysis (EFA). The analysis was performed using Principal Axis Factoring (PAF) extraction method to identify underlying factor structure of the QoR-N.^13^ The Kaiser-Meyer-Olkin (KMO) measure of sampling adequacy and Bartlett’s test of sphericity were used to determine data suitability for factor analysis. A KMO value of ≥0.80 was considered and a statistically significant Bartlett’s test (p<0.05) was taken as evidence that the correlation matrix was factorable.^13^ The number of factors to retain was determined using Kaiser’s criterion (eigen value>1) and visual inspection of the scree plot for a visible inspection point (“elbow”).^13^ As only one factor was extracted, no rotation was applied. Communalities were examined to determine the proportion of variance explained by the extracted factor. A communality value of ≥ 0.3 was considered acceptable for inclusion in a meaningful factor solution.^13^

Responsiveness of the QoR-N was evaluated by comparing the mean preoperative and postoperative scores using paired t-test. To quantify the magnitude of change, Cohen’s effect size (Cohen’s d) and the Standardized Response Mean (SRM) were calculated.^14,15^

- Cohen’s d was calculated using the formula: d= X_post_ - X_pre_ / SD_pooled_
- SRM was calculated as: SRM= X_post_ - X_pre_ / SD of difference score
- Effect sizes were interpreted as small (0.2), moderate (0.5), or larger (0.8)^14^
- SRM interpreted as negligible (< 0.20), small effect size (0.20-0.49), moderate effect size (0.50-0.79), large effect size ≥0.80^15^

## RESULTS

Of the 216 (100%) patients approached, all completed the preoperative QoR-N questionnaire. The postoperative QoR-N questionnaire was completed by 200 (92.59%) patients. Among the 16 (7.41%) patients excluded from the postoperative assessment, 4 (25%) were transferred to the intensive care unit, 9 (56.25%) were lost to follow-up, and 3 (18.75%) refused to answer. None of the patients reported a score of 0 or 120 on the QoR-N, indicating the absence of floor or ceiling effects. The demographic and clinical profiles of the 200 (92.59%) patients who completed both assessments are presented (Table 1). No complications were reported in the first 24 hours following surgery. The median time to postoperative assessment was 23 (IQR: 20-25) hours.

The time required to complete the preoperative questionnaire was 170.46±12.08 seconds and 140.85±15.67 seconds for the postoperative questionnaire. Recovery was graded as poor by 48 (24%) patients. Of the total patients, 135(67.50%) were female and 157(78.50%) were classified as ASA physical status I. The majority underwent intermediate surgery 137(68.50%), with general surgery performed in 109(54.50%) cases. The median age was 40.50 years (IQR: 31-75) (Table 1).

**Figure 1:**
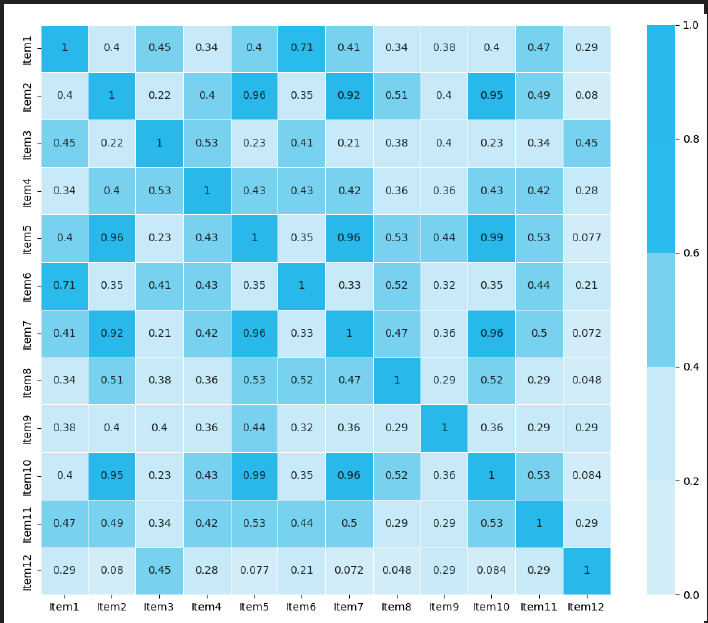
Heatmap of correlation coefficients between individual items of QoR-N (n=200).

Interitem correlation matrix of QoR-N shows Item 2, Item 5, Item 7, and Item 10 exhibit very high correlations among each other, ranging from 0.915 to 0.989, indication they are highly related. Additionally, Item 6 and Item 1 show a notable correlation of 0.707. Cronbach’s alpha was 0.890, mean interitem correlation was 0.413, split half reliability was 0.940 and Standard Error of Measurement was 2.790 (Figure 1).

**Table 1:** Demographic and clinical characteristics of the participants of the Validation of Quality of Recovery-15 questionnaire (n=200).

Characteristic	n(%)
Gender	
Male	65(32.50)
Female	135(67.50)
ASA physical status	
I	157(78.50)
II	43(21.50)
Comorbidities	
Hypertension	11(5.50)
Hypertension + Thyroid	4(2.00)
Hypertension + Diabetes	3(1.50)
Hypertension + Diabetes + Thyroid	2(1.00)
Diabetes	6(3.00)
Extent of surgery	
Minor	12(6.00)
Intermediate	137(68.50)
Major	51(25.50)
Type of surgery	
Urological	33(16.50)
ENT	27(13.50)
General surgery	109(54.50)
Gynecological	31(15.50)

ENT: Ear, Nose, Throat department; ASA: American Society of Anesthesiologists American QoR: score: Quality of Recovery Score

**Table 2:** Change in Preoperative and Postoperative QoR-N Scores (n=200).

Item	Preop QoR-N score (mean)	Postop QoR-N score (mean)	Preop and postop QoR-N score difference	SRM	Cohen’s d	% change from baseline
1	9.43	7.65	-1.78	-2	-1.47352	-18.875
2	9.44	7.83	-1.61	-1.25781	-1.55554	-17.055
3	9.98	8.43	-1.55	-1.86747	-2.51675	-15.531
4	9.83	8.74	-1.09	-1.18478	-1.63951	-11.088
5	9.04	7.87	-1.17	-1.04464	-1.17965	-12.942
6	8.7	6.55	-2.15	-2.19388	-2.14887	-24.712
7	7.65	7.84	0.2	0.188679	0.20491	2.483
8	6.23	9.07	2.83	1.626437	1.805627	45.585
9	7.4	7.06	-0.34	-0.15179	-0.22558	-4.594
10	8.5	7.88	-0.61	-0.28372	-0.46265	-7.294
11	9.34	8.52	-0.82	-0.49697	-0.72511	-8.799
12	9.53	7.63	-1.9	-1.31944	-1.81288	-19.937
Total	105.03	95.04	-9.99	-1.39136	-1.39674	-9.511

Cohen’s d= mean change in score divided by the baseline (preoperative) SD; SRM (Standardized Response Mean) = mean change in score divided by its SD

Construct Validity assessment showed a positive correlation of the VAS score for overall recovery with postoperative QoR-N (correlation coefficient 0.299, p = 0.01). In criterion validity, postoperative QoR-N score correlated with age of the patient (correlation coefficient: -0.021, p = 0.77) and duration of surgery (correlation coefficient: -0.22, p = 0.75). The postoperative QoR-N score among ASA PS grade I and II patients was compared (Mann-Whitney U Test, U = 3117, p = 0.44). Postoperative QoR-N score was compared across minor, intermediate, or major surgery (Kruskal-Wallis test: chi-square = 3.84, p = 0.146). QoR-N scores were lower in females as compared to males (Mann-Whitney U Test, U = 3517.5, p = 0.02). Correlation between QoR-N scores and items of physical and mental well-being were 0.953 (p = 0.00) and 0.865 (p = 0.00), respectively.

Structural validity was assessed by exploratory factor analysis. The Kaiser-Meyer-Olkin (KMO) measure of sampling adequacy was 0.859. Bartlett’s Test of Sphericity was significant (chi-square = 2443.343, p < 0.001). Kaiser’s criterion and the scree plot supported extraction of a single factor, therefore no rotation was applied. Item9 and item 12 had communality less than 0.3 (0.230 and 0.051 respectively). Mean preoperative and postoperative QoR-N scores were 105.0 (SD 5.6) and 95.0 (SD 8.4), respectively. The difference had a p value ≤ 0.01. QoR-N showed an effect size (Cohen’s d = 1.40) and responsiveness (Standardized Response Mean = 1.39) (Table 2).

The recruitment rate was 100%, with a completion rate of94.33%. No floor or ceiling effects were observed. The mean time taken to complete the preoperative questionnaire was 170.46 ± 12.08 seconds, while the mean time for the postoperative questionnaire was 140.85 ± 15.67 seconds.

## DISCUSSION

This study validated the Nepalese version of QoR-15. There was a decline in overall QoR-N score by 9.99 points from preoperative to postoperative (105.03 to 95.04). All the 12 Items were affected by surgery and anesthesia. Individual Item changes ranged from -24.7% (Item 6) to + 45.6% (Item8). Most Items showed a decrease in postoperative scores. The Item 6 (can resume daily activities like before) exhibited the largest decline, highlighting the significant impact of surgery on patients’ functional recovery. Only two Items, Item 7 (feeling of good health) and Item 8 (feeling anxious) showed an increase in postoperative scores reflecting patient relief and reduced aniticapatory stress once the surgical procedure is completed. The total score of QoR-N showed a standardized response mean of -1.39 and Cohen’s d for total score change of -1.40, both well above the threshold of ≥0.8, suggesting a very strong ability to detect a clinically important change in QoR.^14,15^ The negative values reflect the expected decline in overall recovery quality soon after surgery.

The inter-item correlation matrix revealed considerable variability in the strength of association among 12 postoperative items of the QoR-N. Item 10, which evaluates presence of severe pain deonstrated a high positive correlation with Item 2, (patient’s ability to enjoy food; r=0.95), item 5 (receiving support from doctor and nurses; r=0.99), and item 7 (feeling of general well being; r=0.96). Similarly correlation between item 1 and item 6 was high (0.707). This may be due to the interdependence among these items in a postoperative setting where presence of severe pain can be strongly linked with appetite, attention of caregivers and feeling of general well being. On the other hand, some item pairs, especially those involving item12, showed weak or negligible correlations. Similar pattern of weak correlation was reported in other language adaptation of QoR-15.^16,17^ Despite these inconsistencies among specific items, an acceptable mean inter item correlation combined with Cronbach’s alpha and split-half reliability ( 0.890 and 0.940 respectively), suggest that the QoR-N maintains good internal consistency at the aggregate level. The precision of the QoR-N was further supported by SEM of 2.79. This finding aligns closely with the Turkish version of the QoR-15, which reported an SEM of 2.8, and similarly interpreted it as acceptable for clinical use.^18^ However, careful consideration of item redundancy in future refinements may further optimize the scale without compromising content coverage.

Due to the busy clinical environment, the participants were assessed by a single interviewer per time point. Also, in the postoperative period the participants were interviewed only once. So, test -retest reliability, inter-rater and intra-rater reliability of QoR-N could not be evaluated in the present study. Several previous QoR-15validation studies, also refrained from conducting test-retest analyses postoperatively due to practical concerns.^17^

Among 216 patients approached, all agreed to participate in the study. However, only 200 (96.6%) completed the postoperative questionnaire. The feasibility of QoR-N was demonstrated by a recruitment rate of 100% and a completion rate of 92.59%. This high completion rate is consistent with previous studies on QoR-15 and reflects the clarity and acceptability of the tool. ^18,19^ The mean preoperative QoR-N score was 105.0 (SD5.6) and the mean postoperative score was 95.0 (SD 8.4). There was a statistically significant difference in the mean preoperative and postoperative QoR-N score. The study sample had a median age of 40.5 years (IQR 31-75), representing a broad range of adult patients undergoing elective surgery. Female participants predominated, accounting for 67.5%, while males comprised 32.5% of the study population. Most patients were classified as ASA physical status I (78.5%), with the remaining 21.5% in ASA Physical Status II, indicating a predominantly healthy population with mild systemic disease. Surgical procedures varied by complexity, with 6% minor, 68.5% intermediate, and 25.5% major surgeries, reflecting a mix of relatively low to moderate-risk surgeries. The most common specialities were general (54.5%), followed by urology (16.5%), gynecology (15.5%) and ENT (13.5%). Median surgery duration was 75 minutes (IQR 60-110), consistent with intermediate surgical cases. The demographic and clinical characteristics suggest that our sample is diverse in age, sex, and surgical procedure types but relatively healthy in terms of systemic comorbidity. This diversity, supports the generalizability of our findings to the broader surgical population.

The correlation between the duration of surgery and the total QoR-15 was not statistically significant (p=0.75). This finding contrasts with earlier study, where longer surgery duration was often associated with poorer recovery scores.^3^ Similarly, no correlation was found in postoperative QoR-N score and age of the patient. The postoperative QoR-N scores were not statistically different in ASA physical status I and II patients and extent of surgery. Our study population belonged to lower ASA physical status (I and II), with no patients in ASA Physical Status III. In the English version 46% of the patients were of higher ASA Physical Status (III and IV).^3^ Number of minor, intermediate and major surgeries in our study was 12 (6%), 137 (68.5%) and 51 (25.5%). Whereas, in the English version 50% of the surgeries were major.^3^ Disparity in the ASA physical status of study population and extent of surgery in our study might have contributed to the non significant relationship of these variables with QoR-N scores. Some cross -cultural validations, such as the Arabic and Portugese versions, have reported similar non significant correlations, suggesting that the perceived quality of recovery may be influenced more by contextual, cultural, or institutional are factors rather than by surgery specific variables _alone._^20,21^

There are a few limitations in our study. The study was conducted at a single tertiary care hospital in Nepal, which may limit the generalizability of the findings to other settings, such as rural or smaller healthcare facilities. The majority of surgeries were of intermediate complexity, limiting the ability to generalize to patients undergoing more complex or high-risk procedures. The QoR-N questionnaire was investigator directed. Test-retest, inter rater and intra rater reliability could not be assessed due to the time and resource constraints.

## CONCLUSIONS

The Nepalese version of the QoR-15 questionnaire demonstrates good psychometric properties. The tool is suitable for assessing postoperative recovery in Nepali-speaking surgical patients. However, we recommend validating the tool in diverse clinical settings, including rural hospitals, and in high-risk and complex surgeries and assessing recovery over an extended period for better understanding of long-term recover.

## Data Availability

The data are available from the corresponding author upon reasonable request
